# Helping rural women in Pakistan to prevent postpartum hemorrhage: A quasi experimental study

**DOI:** 10.1186/1471-2393-12-120

**Published:** 2012-10-30

**Authors:** Ali Mohammad Mir, Abdul Wajid, Sadaf Gull

**Affiliations:** 1 Director of Programs, Population Council, Islamabad, Pakistan; 2 Program Manager, Population Council, Islamabad, Pakistan; 3 Program Officer, Population Council, Islamabad, Pakistan

**Keywords:** Postpartum hemorrhage, Home-based deliveries, Rural communities, Traditional birth attendants, Misoprostol, Pakistan

## Abstract

**Background:**

According to the Pakistan Demographic and Health Survey from 2006–2007, the maternal mortality ratio in rural areas is 319 per 100,000 live births. Postpartum hemorrhage is the leading cause of maternal deaths in Pakistan. The objectives of the study were to document the feasibility of distribution of misoprostol tablets by community-based providers mainly traditional birth attendants and acceptability and use of misoprostol by women who gave birth at home.

**Methods:**

A quasi-experimental design, comprising intervention and comparison areas, was used to document the acceptability of providing misoprostol tablets to pregnant women to prevent postpartum hemorrhage in the rural community setting in Pakistan. Data were collected using structured questionnaires administered to women before and after delivery at home and their birth attendants.

**Results:**

Out of 770 women who delivered at home, 678 (88%) ingested misoprostol tablets and 647 (84%) ingested the tablets after the birth of the neonate but prior to the delivery of the placenta. The remaining women took misoprostol tablets after delivery of the placenta. Side effects were experienced by 40% of women and were transitory in nature. Among women who delivered at home, 80% said that they would use misoprostol tablets in the future and 74% were willing to purchase them in the future.

**Conclusions:**

Self-administration of misoprostol in the home setting is feasible. Community-based providers, such as traditional birth attendants and community midwives with proper training and counseling, play an important role in reducing postpartum hemorrhage. Proper counseling and information exchange are helpful for introducing new practices in resource-constrained rural communities. Until such a time that skilled birth attendance is made more universally available in the rural setting, alternative strategies, such as training and using the services of traditional birth attendants to provide safe pregnancy care, must be considered.

## Background

Pakistan is among the few countries in South Asia that continues to have poor reproductive health indicators. Although there have been some improvements in recent years, maternal mortality figures in Pakistan remain high. Because of limited access to health facilities, approximately two-thirds of all births (61%) take place at home, where a traditional birth attendant (TBA or dai) or a family member usually attends the delivery [1].

Among the major causes of maternal deaths, postpartum hemorrhage (PPH) is the most common. According to the Pakistan Demographic and Health Survey of 2006–2007, 27.2% of maternal deaths were caused by PPH [1].

To prevent PPH, injectable oxytocin is recommended at the time of delivery as per routine. Oxytocin requires storage in a cool temperature (5–25°C), and can only be kept outside these temperatures for short durations. To administer injectable oxytocin, a skilled provider trained in safe injection practices and the availability of sterile syringes and needles are required [2].

Misoprostol, a prostaglandin E1, acts as an alternative uterotonic to the injectable drug oxytocin and is available in tablet form. Misoprostol has been found to be effective in controlling, as well as preventing, PPH [3,4]. Misoprostol is inexpensive, does not require any special conditions for its storage, and its tablet form can be easily administered by a community health care provider, a TBA, and even the woman herself who has just delivered.

Results from a study in Egypt recommend the use of misoprostol, even in the hospital setting if there are staff shortages, refrigeration issues and/or high caseloads, because misoprostol offers the advantage of a one-drug regimen, stability, and easy administration, compared with injectable uterotonics [5]. Misoprostol causes occasional side effects, which include shivering and fever [4,6-8]. The use of misoprostol has been recommended by the United States Pharmacopoeia Expert Committee for the Prevention of PPH, especially in settings where injectable uterotonic drugs are not available [9]. Misoprostol has also been added to the WHO essential drugs list [10].

In Pakistan, two research studies examined misoprostol use, conducted in hospital and community settings. Both studies were carried out by Aga Khan University. One of the studies was a hospital-based treatment-placebo-controlled trial in which 600 μg of misoprostol or a matching placebo was given sublingually in addition to standard PPH treatment with injectable oxytocics [6]. The results supported adjunct use of misoprostol, but there were no significant differences between the intervention (oxytocin and misoprostol) and comparison (oxytocin-placebo) groups. The trial had to be terminated because the PPH rate was low. Another community-based placebo-controlled trial was conducted in Chitral in the northern areas where TBAs were used to provide 600 μg of misoprostol orally to women shortly after they gave birth. The results showed that oral misoprostol reduced the rate of PPH by 24% compared with the placebo. Referrals for higher-level care during the immediate postpartum period were similar following administration of misoprostol (1.7%) [11].

The Population Council, as a consortium partner of the USAID-sponsored Pakistan Initiative for Mothers and Newborns (PAIMAN), undertook an operations research project in two districts of Pakistan to assess the feasibility of home administration of misoprostol for the prevention of PPH in rural Pakistan. The purpose of undertaking the study was based on the premise that such an intervention has the potential to provide a model for more effective safe motherhood interventions in rural areas.

The current study had the following objectives: to document the feasibility of distribution of misoprostol by community-based providers, including TBAs (called dais in Pakistan), in a home setting; to assess the acceptability and use of misoprostol by pregnant women for the prevention of PPH when administered at home; to identify the common side effects of misoprostol; and to determine the reduction in demand for referral due to PPH after oral ingestion of misoprostol.

## Methods

A quasi experimental design was used comprising intervention and comparison areas (see below for more detail). The study was conducted in the districts of Dadu in Sindh Province and Khanewal in Punjab from October 2009 to September 2010. Both districts were focus districts of the PAIMAN project.

### Participants and data collection

Pregnant women living in the selected union councils of Khanewal and Dadu districts who were 15–49 years of age and intended to deliver at home, were willing to participate in the study, and would be delivering during the study period were included in the study. Selection criteria of eligible pregnant women varied in different villages depending on when registration occurred. When registration started in the beginning of the study, women in their second and third trimesters were included, while in villages where registration was performed later in the study, women only in their third trimester were included. The date of delivery was estimated using the Expected Date of Delivery Calculator wheel.

### Sample size/sample size estimation

Currently, the PPH rate is estimated to be 11% of live births globally [12]. For a developing country, such as Pakistan, we expected this to be in the range of 10-15%. We expected that this rate could be reduced by half (to 5–7%) with the introduction of misoprostol. We also assumed that half of the women with PPH would be referred irrespective of the project area. Therefore, in the comparison areas, approximately half of the women with PPH (7%) would be referred, while in the intervention areas where the PPH rate was expected to be 5–7%, referral would be reduced by half (3%). We calculated the sample size with α at 5% and the power of the study at 80%. We determined that a minimum of 780 deliveries in each of the two groups was required to test the difference of a 50% reduction in reported PPH [13].

Six union councils in Dadu district and seven in Khanewal closest to the selected referral health facilities were included in the study to meet the required sample size. In Khanewal district, seven union councils were selected, with four to serve as intervention areas and three as comparison areas. In Dadu, six union councils were selected, with three each to serve as intervention and comparison areas. A union council has a population of approximately 25–30,000 people. Union councils were selected to avoid diffusion among the intervention and comparison areas.

### Study phases

#### I. Creating community awareness regarding study objectives

The study consisted of two phases. The first phase involved community awareness and family education, through group interaction and individual counseling with pregnant women and their family members. These were carried out in separate sessions. At the time of registration, each identified pregnant woman and her family members (in both the comparison and intervention areas), were given a briefing on birth preparedness and obstetric danger signs using a pictorial booklet. For women and their attendants in the intervention areas, the briefing also included information regarding the use of misoprostol, including correct timing, dosage and its side effects. One month prior to the expected date of delivery, the women were interviewed to assess retention of knowledge. Fifteen days before the expected delivery date, the women were again briefed on the information provided earlier along with the information on referral facilities. During this visit, clean delivery kits were given to the women in both areas. In the intervention areas, the kits also contained three tablets of 200 μg of misoprostol each, along with a pictorial instructional leaflet on its use. For this research, Venture Strategies Innovations (VSI) donated 6,000 misoprostol tablets.

The 566 birth attendants identified by the pregnant women expected to carry out the deliveries were given a 1-day interactive training in 28 batches comprising nearly 20 birth attendants per batch. This training was spread over a period of 2 weeks. All birth attendants were also given clean delivery kits.

#### II. Data collection from postpartum women and their birth attendants

In the second phase, data collection was carried out immediately after delivery from women who had delivered, their birth attendants, and relatives present at the time of delivery. Separate consent forms were used for each of the questionnaires. Written informed consent was obtained from all the study participants in both the intervention and comparison areas after providing them full information about the study. Social scientists read the informed consent to all the illiterate women who were unable to read and sign. For all such illiterate women, the informed consent form was verified by any of their relatives present at that time.

### Safety management

Trained lady health visitors were identified in the area to provide back-up support in case of emergency. Information was provided to families regarding referral facilities where 24-hour emergency services were available and could be reached within 1 hour’s travelling time. The identified referral facilities were the public sector Civil Hospital in Dadu and Tehsil Hospital Mian Channu in Khanewal.

#### Data management and analysis

The data were coded and entered using CSPro (version 3.2) and SPSS (version 14). All data and forms were stored in locked cabinets at the Population Council office in Islamabad. Results were summarized using frequency distributions and cross tabulations. Bivariate analysis based on intervention and comparison areas were used to assess differences between indicators. Statistical significance was established at a P value of 0.05.

### Ethical consideration

This study was conducted with the approval of the National Maternal, Neonatal and Child Health (MNCH) Program, Ministry of Health, Government of Pakistan. Ethical clearance was accorded by the Institutional Review Board of the Population Council.

## Results

**Figure 1:**
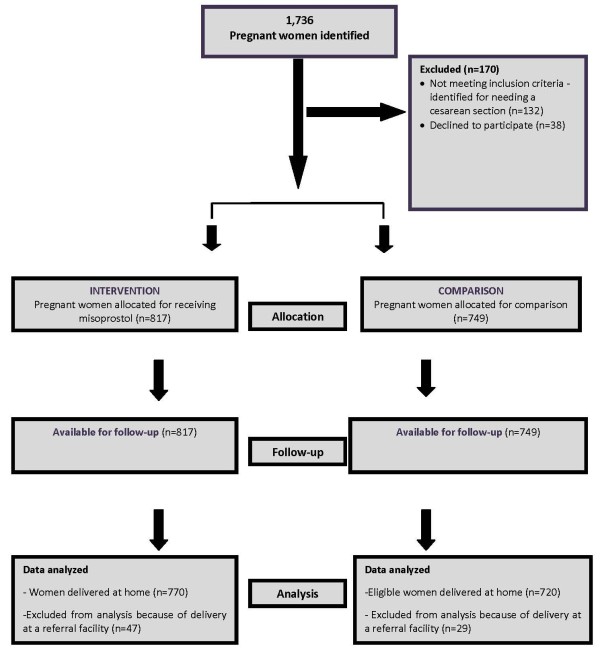
CONSORT flow diagram: Study profile.

A total of 1736 women were identified for participation in the study (Figure 1). Out of 890 pregnant women assigned to the intervention areas, 18 (2%) refused to participate in the study. Another 55 were deemed ineligible as they had been recommended to have facility-based delivery by caesarean section. Forty-seven women were referred to a health facility during labour for obstetric complications, mainly because of prolonged labor, and were not included in the final analysis. In total, 770 women who delivered at home were considered eligible for taking misoprostol. In the comparison area, out of the 846 eligible women, 20 (2%) refused to participate in the study, and 77 women who were identified for cesarean sections were deemed ineligible. Twenty-nine women were referred for obstetric complications, and therefore, were excluded from the final analysis, leaving 720 eligible women [9]. 

### Characteristics of the women

The characteristics of women in both the intervention and comparison areas were almost similar. The mean age was 28 ± 5.7 years in the intervention areas and 28 ± 6.0 years in the comparison areas. The mean number of live births was 3.0 ± 2.8 in the intervention areas and 2.8 ± 2.6 in the comparison areas. Nearly half of the respondents in both areas had made at least one antenatal visit. Nearly two-thirds of the women in both the intervention and comparison areas were uneducated (73% and 78% respectively).

### Knowledge of respondents

When the study participants were interviewed 1 month before their expected delivery date to ascertain how much of the information they had retained from the initial briefing provided to them, 95% of the women in the intervention areas knew that tablets taken orally could prevent PPH. Approximately 52% of these women were even able to correctly recall that the name of the drug was misoprostol. The other women, while aware of the misoprostol tablets, could not mention the generic name.

With respect to knowledge regarding appropriate timing for referral, 93% of respondents in the intervention area and 89% in the comparison areas knew that in case of heavy bleeding (two or more soaked cloth pads), women must be referred to a facility within 1–2 hours.

### Misoprostol use: safety, acceptability and referral

#### Safety

Among the 770 eligible women in the intervention areas who delivered at home, 678 women (88%) took the misoprostol tablets provided to them in the clean delivery kits (Table 1). Among these, 647 (84%) took the misoprostol tablets correctly, i.e., three misoprostol tablets ingested after the birth of the neonate, but prior to the delivery of the placenta. The remaining 31 women took the misoprostol tablets after the delivery of the placenta. None of the women took the misoprostol tablets before delivery. However, 92 women (12%) did not take the misoprostol tablets (Table 2). More than half of these respondents (49 cases) said that they forgot to take the misoprostol tablets. Family opposition as a reason for not taking the tablets was considered to be negligible, with only 13 cases reporting hindrance from relatives. Out of these, only one woman reported that her husband opposed the use of misoprostol.

**Table 1:** Use of misoprostol in intervention areas

	**Number of respondents (n)**	**Percentage (%)**
Eligible women who delivered at home	770	100
Women who used misoprostol tablets	678	88
-Before the baby was born	0	0
-Took 3 misoprostol tablets after delivery of the neonate and before delivery of the placenta	647	84
-After the placenta was delivered	31	4

**Table 2:** Main reasons for not taking misoprostol (n=92)

**Reasons**	**Number of respondents (n)**	**Percentage (%)**
Self-reservation	11	11.9
Family members opposed	13	14.3
Forgot	49	53.2
TBA/dai opposed	11	11.9
Others	8	8.7
Total	92	100.0

#### Side effects

Of the 647 women who took misoprostol tablets correctly (correct dose and correct time), 40% reported that they had experienced side effects (Table 3). The majority of women reported suffering from shivering/chills, followed by fever and nausea. However, in all of these cases, the symptoms were transient and did not require referral for specialized care. Among the 31 women who took the tablets incorrectly, 17 experienced side effects, the most common being shivering reported by 13 women.

**Table 3:** Use of misoprostol and reported side effects

	**Number of respondents (n)**	**Percentage (%)**
**Women who took misoprostol correctly and reported side effects**	260	40
**Women who took misoprostol incorrectly and reported side effects**	17	55
**Women who took misoprostol and did not report side effects**	387	60
**Women who took misoprostol incorrectly and did not report side effects**	14	45
Major side effects experienced by those who took misoprostol correctly* (n=260)		
Shivering/chills	178	69
Fever	68	27
Vomiting	23	9
Nausea	43	17
Abdominal pain	42	16
Major side effects experienced by those who took misoprostol incorrectly* (n=17)		
Shivering/chills	13	77
Fever	8	47
Vomiting	3	18
Nausea	2	12
Abdominal pain	3	18
Others	2	12

*Multiple response variable.

#### Acceptability

Nearly 80% of the eligible women who delivered at home said that they would use the tablets in the future. Seventy-four percent of women said that they would also be willing to purchase the misoprostol tablets in the future, and 80% of women said that they would recommend the drug to others.

Of those birth attendants in the intervention areas who had administered misoprostol tablets, almost all of them (98%) said that they would recommend the tablets to other clients.

### PPH and referrals

The 770 women in the intervention areas and 720 women in the comparison areas who delivered at home were asked about their perception of postpartum blood loss. A total of 40 women (5%) in the intervention areas and 82 women (11%) in the comparison areas reported perceiving heavy postpartum bleeding short of PPH. This heavy bleeding was characterized as blood loss perceived as more than normal menstrual bleeding that was not continuous and without having any clots. Women in the intervention areas were less likely to perceive heavy postpartum bleeding (RR: 0.45; 95%CI: 0.31-0.65) (Table 4). Similarly, women who had taken misoprostol correctly were less likely to report having excessive bleeding after delivery (RR: 0.43; 95%CI: 0.29-0.64) (Table 5).

**Table 4:** Distribution of women according to perception of heavy postpartum vaginal bleeding

**Area**	**Perception of heavy bleeding**					
	**Not perceived (n)**	**Percentage (%)**	**Perceived (n)**	**Percentage (%)**	**RR**	**95% CI**
Intervention	730	94.8	40	5.1	0.45	0.31-0.65
Comparison	638	88.6	82	11.3		

**Table 5:** Distribution of women who perceived heavy postpartum vaginal bleeding, by intake of misoprostol tablets

**Area**	**Perception of heavy bleeding**					
	**Not perceived respondents (n)**	**Percentage (%)**	**Perceived respondents (n)**	**Percentage (%)**	**RR**	**95% CI**
Intervention (incorrectly taken)	29	93.5	2	6.5	0.56	0.14-2.19
Intervention (correctly taken)	615	95.1	32	4.9	0.43	0.29-0.64
Not taken	86	93.5	6	6.5	0.57	0.25-1.27

The comparison area is the reference.

CI: Confidence Interval.

Women were identified as suffering from PPH and requiring referral by the birth attendant present at the time of delivery if two or more cloth pads were soaked with blood within 1 hour after delivery.

In the intervention areas, three women (0.4%) suffered from PPH that required referral to a higher-level facility. Out of these, two women had ingested misoprostol; 1 of the women who had not ingested misoprostol later died upon reaching a health facility. Two additional facility deaths were also reported in women from the intervention area; these were due to ante-partum hemorrhage and eclampsia. In the comparison areas, five (0.6%) women required referral for the management of PPH; of these women, two died upon reaching the facility. In addition, there were three more facility-based maternal deaths due to complications of the upper respiratory tract, uterine rupture, and exacerbation of pre-existing cardiac disease. The cause of death was ascertained through verbal autopsies conducted with hospital staff where the women had been referred. In both the intervention and comparison areas, no maternal deaths took place within the home setting.

## Discussion

Pakistan is one of the few countries in the region where lowering maternal mortality continues to be a challenge. Approximately 67% of the population resides in rural areas, often away from health facilities, which are mostly concentrated in urban areas. Because of the paucity of emergency obstetric care facilities, encouraging institutional deliveries at present is not a viable option for improving maternal outcomes. To help rural women safely deliver, the government of Pakistan launched the national MNCH program in 2006. The aim of the program is to train and place community midwives (CMWs) in rural areas; however, the deployment of these workers has been limited [14]. The CMWs still require some time before they are fully established and recognized as a viable and worthwhile alternative to the existing traditional birth attendants. An interim strategy would be to improve the safety of home-based deliveries. The results from this study showed that through proper counseling and training, well-entrenched behaviors and practices of women, their families, and traditional providers could be altered. This is because our results showed that a high percentage of women agreed to adopt a new practice that was supported by the TBAs. A study conducted in Tanzania also showed that TBAs can safely use misoprostol for the treatment of PPH [3].

Our study results are similar to the results reported in earlier studies that assessed the feasibility of administering misoprostol within the home setting. For example, in a study carried out in West Java, Indonesia, more than three quarters of women in the intervention area used oral misoprostol tablets [15], whereas in a community-based study in Afghanistan, it was found that nearly 70% of the identified pregnant women used misoprostol [2]. Despite the transient side effects experienced by women who took the drug, this did not prevent 80% of the women from saying that they would take the drug for future deliveries.

We found that three important factors contributed to the acceptability and use of misoprostol tablets. First and foremost was community awareness, which, to a great extent, was facilitated by the local public sector community-based lady health workers who helped in targeting pregnant women and their family members. Second, and also part of community awareness, were discussions with community elders and influential people. Third, counseling sessions provided accurate and credible information directly to the women, their family members, husbands, and birth attendants using culturally appropriate information, education and communication materials.

After providing information regarding PPH and misoprostol use, we interviewed the women close to their time of delivery to determine what information they had retained. An encouraging finding was that the majority of respondents knew the time limit by which a woman suffering from PPH needs to reach a facility.

In the current study, the high number of referrals in the intervention and comparison areas can be attributed to awareness created about obstetric danger signs in the families and birth attendants. However, the high number of maternal deaths of women, at facilities other than the specified referral facility, highlights the dire need for improving the quality of emergency obstetric care within the public and private sectors, especially in rural areas. Based on our results, we can recommend that the continuum of care approach starting from the home until reaching a facility is the only way to reduce maternal deaths in the rural setting.

The PPH rate in our study was lower than what we had expected. This could be an underestimate as we were not actually measuring blood loss. In a randomized control trial conducted in Karachi, Pakistan, in four hospitals, the occurrence of PPH was also found to be low, suggesting that previous estimates had been based on an “over-estimation” of postpartum blood loss by hospital staff due to “incorrect measurement, resulting in misdiagnosis, unnecessary treatment, and prolonged hospitalization” [6].

The results from this study provide a useful addition to the literature on the feasibility of home-based administration of misoprostol for the prevention of PPH in resource-constrained settings.

## Conclusions

This study found that misoprostol administration within the home setting in rural communities is possible, and proper counseling and information exchange can introduce new practices in rural communities.

## Abbreviations

CMW: Community midwife; LHW: Lady health worker; MNCH: Maternal neonatal and child health; PAIMAN: Pakistan initiative for mothers and newborns; PPH: Postpartum hemorrhage; SPSS: Statistical package for the social sciences; TBA: Traditional birth attendant; WHO: World health organization.

## Competing interests

The authors declare that they have no competing interests.

## Authors’ contributions

AMM was involved in conceptual design for this study, interpretation of data, drafting of the manuscript and critically revising for intellectual content. AW was involved in design of conceptual structure. He later contributed to data analysis, interpretation and was involved in drafting the paper. SG was involved in acquisition of data, analysis and drafting the paper. All authors read and approved the final manuscript.

## Pre-publication history

The pre-publication history for this paper can be accessed here:

http://www.biomedcentral.com/1471-2393/12/120/prepub
